# Nutraceutical Approaches of Autophagy and Neuroinflammation in Alzheimer’s Disease: A Systematic Review

**DOI:** 10.3390/molecules25246018

**Published:** 2020-12-18

**Authors:** Reinhard Gruendler, Berit Hippe, Vesna Sendula Jengic, Borut Peterlin, Alexander G. Haslberger

**Affiliations:** 1 Department of Pharmacology and Toxicology, University of Vienna, A-1090 Vienna, Austria; reinhard@gruendler.cc; 2 Department of Nutritional Sciences, University of Vienna, A-1090 Vienna, Austria; berit.hippe@univie.ac.at; 3 HealthBioCare, Nußdorfer Street 67, A-1090 Vienna, Austria; vesna.sendula@gmail.com; 4 Rab Psychiatric Hospital, 51280 Kampor, Croatia; borut.peterlin@kclj.si

**Keywords:** neuroinflammation, cognitive decline, epigenetic, autophagy, EGCG, fisetin, spermidine

## Abstract

Aging and the emergence of age-associated illnesses are one of the major challenges of our present society. Alzheimer’s disease (AD) is closely associated with aging and is defined by increasing memory loss and severe dementia. Currently, there are no therapy options available that halt AD progression. This work investigates three hallmarks of the disease (autophagy, neuroinflammation, and senescence) and systematically analyzes if there is a beneficial effect from three substances derived from food sources, the so called “nutraceuticals” epigallocatechin gallate, fisetin, and spermidine, on these hallmarks. The results imply a positive outlook for the reviewed substances to qualify as a novel treatment option for AD. A combination of nutraceutical substances and other preventive measures could have significant clinical impact in a multi-layered therapy approach to counter AD.

## 1. Introduction

According to US Centers for Disease Control and Prevention (CDC) and the US National Institutes of Health (NIH), subjective cognitive decline (SCD) is the self-reported experience of worsening or more frequent confusion or memory loss. It is a form of cognitive impairment and one of the earliest noticeable symptoms of Alzheimer’s disease (AD) and related dementias. SCD can have implications for living with and managing chronic disease or performing everyday activities like cooking or cleaning. Because SCD is self-reported, it does not imply a diagnosis of cognitive decline by a health care professional.

Cognition is a combination of processes in the brain that includes the ability to learn, remember, and make judgments. When cognition is impaired, it can have a profound impact on an individual’s overall health and well-being. Cognitive decline can range from mild cognitive impairment to dementia, a form of decline in abilities severe enough to interfere with daily life.

The prevalence of SCD is 11.1%, or 1 in 9 adults. The prevalence of SCD among adults aged 65 years and older is 11.7% compared to 10.8% among adults 45–64 years of age. The prevalence of SCD is 11.3% among men compared to 10.6% among women. The prevalence of SCD differs among racial/ethnic groups, 10.9% of Whites report SCD compared to 12.8% of Blacks/African Americans, 11.0% of Hispanics, and 6.7% of Asians and Pacific Islanders. Lower prevalence of SCD is reported in adults with more years of formal education [1].

AD is the most common form of dementia and causes 60–70% of all dementia cases [2]. Worldwide, an estimated 40 million people—mostly older than 60 years—have dementia, and until at least 2050, this number is said to double at least every 20 years [3]. About 6% of people over 65 years suffer from AD1, which is defined by gradually worsening symptoms, but also includes a long, symptom-less pre-dementia stage, where the underlying pathogenetic processes start to form, often 10–20 years prior to first dementia symptoms [4].

Even after more than 35 years since the definition of AD, the definitive cause and pathogenesis of the disease are still not entirely known. Amyloid β (Aβ)-plaques and neurofibrillary tangles formed by tau protein aggregates have been discovered and linked to the disease. They have proven to be a good source for the exploration of molecular pathogenetic events, but targeting these hallmarks alone has not shown satisfactory results in finding a curative or disease-modifying treatment. Since then, many drug candidates have failed in clinical development—and since 2003, no new AD drug candidates have been approved by the FDA at all [5,6,7,8].

Taking into account available therapy options, novel approaches in the prevention of AD and other neurodegenerative diseases could be necessary. One possible solution could be a combination of easily and cheaply available food-derived substances (so called “nutraceuticals”) with a low profile of adverse reactions, aiming at the multiple involved characteristics of the disease.

AD is defined by several hallmarks besides the formation of Aβ-plaques and tau tangles. Because of their special role in the formation of AD, in this review the processes of autophagy, neuro-inflammation, and senescence are investigated.

## 2. Autophagy and AD

Autophagy describes the cellular process of clearance and recycling of damaged or aggregated molecules and cell structures, e.g., proteins, lipids, or cell organelles. Additionally, it is a cellular form of survival technique that a cell uses if it enters a state of nutrient starvation. The activity of autophagy decreases in aging model organisms, and stopping this decline has been associated with an increased lifespan in those organisms [7].

There are four main pathways that induce autophagy. In this chapter the pathways macroautophagy, microautophagy, chaperone-mediated autophagy, and others are described.

Macroautophagy is the most common type of autophagy, and the most well studied. It is a non-selective proteolytic process in eukaryotes, that clears the cell from damaged cell organelles and long-lived proteins, which are then catabolized [9,10,11].

In recent years, the understanding of macroautophagy and related processes has been amplified with the exploration of several genes that play distinct roles in the process. Additionally, a class of miRNA has been linked to play a role in the crosstalk between autophagy and apoptosis that potentially work as molecular switches between these two intimately connected processes and contribute to the cell fate decision [12].

Microautophagy differs from macroautophagy that the cell debris is enclosed directly in the lysosome, and it does not require autophagosome formation for the delivery of targeted cargos to lysosomes [9,13].

Chaperone-mediated autophagy (CMA) also does not require formation of an autophagosome. The activating pathways also are not fully known, but it is commonly understood as a selective mechanism. Kobayashi [9] describes it as follows: “With cooperation of the cytosolic chaperone Hsc70, other co-chaperones, and the lysosomal receptor LAMP2A, proteins are delivered directly to the lysosomal lumen and degraded for intracellular quality control. Although the effect of CMA on those proteins is limited, it is suggested that the impairment of CMA is tightly linked to the pathogenesis of neurodegenerative diseases and cancer, indicating that CMA is an indispensable pathway in maintaining health”.

Other types of autophagy include mitophagy (selective degradation of mitochondria using autophagous methods) and lipophagy (degradation of lipids). Still, there is ongoing debate in the scientific community about whether there might be even more types or pathways of autophagy [9].

In the healthy human brain, clearance of misfolded and unused proteins is an important factor to maintain normal cerebral functions. In turn, AD models have shown that there is a significantly increased accumulation of Aβ-plaques and tau tangles if autophagy is impaired. In AD, impaired mTOR-dependent and independent pathways contribute to the dysfunction of autophagy [14]. This effect is not limited to this two proteins. In contrast, as autophagous activity declines with ageing, there are more proteins that can misform or create toxic aggregates. Therefore, agents that target a single misfolded protein may be far less effective than drugs that enhance autophagy and clearance of misfolded proteins as a whole [7].

Under normal cellular conditions, the cell enters an autophagous state in an environment of nutrient starvation. Since this is not necessarily an available option, influencing the autophagy-inducing mechanisms is a more viable alternative. As these underlying cascades are relatively complex, targeting autophagy in a possible treatment strategy for AD requires specific knowledge of the involved pathways and stages. Ideally, a combination of drugs or substances that target more than one involved cascade could be most promising to restore normal autophagous activity in AD patients.

Additionally, it has shown that simply increasing autophagous activity alone is not necessarily beneficial, as it leads to accumulation of autophagosomes and undigested autolysosomes which can block axonal trafficking and lead to axonal swelling [7]. Still, there are some promising substances that support targeting autophagy as a trigger of neurodegenerative diseases:

A widely used autophagy activator is rapamycin, which blocks mTOR-C1-kinase activity, which is elevated in the brain of 3xTg-mice (a triple-transgenic mouse model of AD) [15]. Blocking this pathway also reduced Aβ and tau pathology and significantly reinstated cognitive impairment.

Since the mTOR pathway also has functions in several cell processes, such as metabolism and growth, inhibition may involve some harmful side effects. Therefore, more studies are now investigating mTOR-independent pathways to modulate autophagy [10].

Further, studies in transgenic mice have shown that methylthioninium chloride (methylene blue) is a potent inducer of autophagy. It altered the levels of LC3-II, cathepsin D, BECN1, and p62, which are all autophagy indicators that are involved in regulating the underlying pathways. In doing so, formation of tau tangles could be decelerated [16].

Apart from the therapeutic potential that autophagous processes represent, there also is a need to find more accurate AD-associated biomarkers that are able to reliably quantify autophagic activity in human brains.

## 3. Neuroinflammation and AD

Inflammatory processes, especially of the central nervous system and brain (neuroinflammation), appear to be some of the key processes in neurodegenerative diseases and aging as a whole. On one hand, they are an important tool for the organism to help recover from certain diseases. On the other hand, they often contribute to disease progression itself.

In the central nervous system (CNS), microglia cells (part of the innate immune system) control inflammatory processes and usually maintain normal CNS function by surveilling their surrounding microenvironment, maintaining homeostasis and neuronal integrity. Upon arrival of adverse stimuli, they are activated and respond according to the external stimulation factors [17].

Microglial cells behave differently according to the amount and type of activation or damage. Under moderate or transient activation, they act as protective mediators for cells, playing an immune resolving, anti-inflammatory part and supporting their surrounding cells by secreting factors that promote cell renewal (TGF-β, IL-10, Arginase-1, Ym-1, etc.). In this state, they act as neuroprotective mediators. In contrast, when intensive acute or persisting microglial activation occurs, they secrete different pro-inflammatory cytokines (TNF-α, IL-6, IL-1β, COX-2) in combination with reactive oxygen and nitrogen species (ROS, NOS). These are substances that promote neuronal damage, disturb neurotransmitter function, and ultimately lead to irreversible tissue loss.

Toll-like receptors (TLRs) also play an indispensable role in cytokine release and pro-inflammatory processes. They activate and signal their downstream pathway to activate NF-κB and pro-IL-1β, both of which are responsible for neuroinflammation and linked to the pathogenesis of different age-related neurological conditions [18].

It is understood that inflammatory processes and persistent microglial activation contribute to cellular aging—a connection, for which the term “inflammaging” has been found [7]. Especially systemic, chronic, and low-grade inflammation have shown to be significant risk factors for morbidity and mortality in aging individuals. Systemic inflammatory biomarkers (e.g., IL-6, fibrinogen, and C-reactive protein) are associated with a decline in regional cerebral blood flow, cortical thinning, and poorer abilities in learning and memory function. Activated microglial cells also lose their phagocytic ability, which further contributes to accumulation of detrimental molecules, which can play a role in AD and other neurodegenerative diseases [7,19].

Regarding pathogenetic processes, it is generally accepted that chronic neuroinflammation in AD patients is not caused by senile plaques and tau tangles, but has to be understood as an independent process that contributes to AD progression and pathogenesis as much as plaques and tangles [20]. Nevertheless, the two cascades are intertwined, as it has been shown that Aβ-oligomers can activate several receptors on the microglial cell surface, which then release proinflammatory cytokines. The cross-talk of these cascades combined with pathological accumulation of Aβ is a key factor that drives neuroinflammatory responses in AD.

Additionally, it has been shown that the autophagy and neuroinflammation cannot be seen as independent processes. Anti-inflammatory effects are in some cases secondary to autophagy induction, because autophagy itself possesses anti-inflammatory effects that rely in part on inflammasome inhibition [21,22].

## 4. Senescence, Senolysis, and AD

Cellular senescence describes a state in the cell cycle in which the cell permanently arrests its cell cycle and stops dividing. It was first discovered in fibroblasts, which stop dividing after about 50 cell population doublings before entering “replicative senescence”. This “hayflick limit” is a very robust tool of the organism to stop old or damaged cells from accumulating. It paved the way for the exploration of cellular aging and its underlying mechanisms [23]. Hayflick, who discovered this mechanism in 1961, hypothesized that these now nondividing cells were involved in the processes of aging, because they had lost the ability to participate in repairment and regeneration within tissues, which was later confirmed [24]. After activation of senescence, senescent cells need to be cleared from the organism, so that they do not accumulate. This can be achieved by either selectively destroying those (as done by senolytic drugs) or inhibiting their function (senostatic drugs) [25].

Conclusively, senescent cells exhibit the following properties: irreversible replicative arrest, apoptosis resistance, and frequently acquisition of a pro-inflammatory, tissue-destructive senescence-associated secretory phenotype (SASP), where cells produce high levels of inflammatory cytokines, immune modulators, growth factors, and proteases, which in turn facilitates hallmarks of AD such as neuroinflammation [26]. Additionally, they prevent their apoptotic clearance by using so called pro-survival senescent cell anti-apoptotic pathways (SCAPs)—often targeted in the development of senolytic drugs [27,28].

Senescence can be induced by a variety of intra- and extracellular factors of cellular stress including abnormal cellular growth, oxidative stress, and autophagous processes. DNA damage, reactive oxygen species (ROS), strong mitogenic signals, depletion of certain tumor suppressors, or mitotic stress also induce senescence [24]. In a normal cell cycle and cellular aging, cells stop replicating after about 50 divisions. This is caused by shortening of telomeres (repeating TTAGGG nucleotide sequences at the end of chromosomes), which occurs because DNA polymerases are not able to completely replicate these sequences. As critically short telomeres can lead to chromosomal instability and tumor formation, the cell enters a state of cell cycle arrest and stops dividing [29].

Disregarding the initiating circumstances, entering the senescent state is coordinated by the p53/p21 and the p16 tumor-suppressive pathways. Uncapped telomeres and DNA double-strand breaks activate a DNA damage response that leads to stabilization of p53 through posttranslational phosphorylation by ATM and ATR serine/threonine protein kinases or by blocking of p53 degradation. Transcription of the cyclin-dependent kinase inhibitor (CDKi) p21 occurs upon p53 stabilization, leading to an initial arrest of the cell cycle. After this initial transient arrest, permanent arrest is controlled by p16INK4A transcriptional upregulation through p38 and/or ERK signaling. Once present, p16INK4A inhibits the activity of both CDK4 and CDK6, thereby leading to RB hypophosphorylation and permanent blockage of S phase entry [24].

Once the cell enters the state of replicative senescence, this cell cycle state is irreversible, and the cell utilizes SCAPs to stay alive [30]. Additionally, a certain phenotype of senescent cells has been identified that releases proinflammatory cytokines [27], which in turn again promote processes involved in neuroinflammation (as discussed above).

The link between senescence and AD-inducing pathways is well documented. In patients with neurodegenerative diseases, various markers of senescence have been observed. Additionally, it has been shown that senescent cells that express the cell cycle inhibitory protein p16 actively drive age-related tissue deterioration and shorten healthy lifespan in mice [31].

In 2018, Bussian et al. [30] found a causal link between the accumulation of senescent cells and cognition-associated neuronal loss. In a mouse model of tau-dependent neurodegenerative disease, they were able to show that p16-positive senescent astrocytes and microglial cells accumulate. Upon clearing these cells, several AD-linked biomarkers improved, including gliosis, hyperphosphorylated tau accumulation, and degeneration of cortical and hippocampal neurons.

The available evidence therefore suggests that there is a link between the cellular mechanism and several age-related diseases such as AD, atherosclerosis, and osteoarthritis [30].

Persisting and accumulating senescent cells not only negatively influence the outlook for neurodegenerative diseases, but have also been identified as a negative factor for other age-related health factors. In mice, clearance of p16-positive cells delayed tumorigenesis and attenuated age-related impairment of several organs including kidney, heart, and fat tissue. In mouse models, knockout of the related genes and subsequent clearance of the senolytic cells increased the healthy life span and occurred without apparent side effects [31]. Whereas gene-knockout is not an option in humans, pharmacological elimination of senescent cells using senolytic drugs could be a promising therapeutic approach. The first senolytic drug (agents that selectively induce apoptosis in senescent cells) was described by Zhu et al. in 2015 [32]. Since then, more potential candidates have been identified. A few notable examples include:

Navitoclax, a small molecule that occupies the inhibitory binding regions of members of the BCL-2 (B-cell lymphoma) family of proteins, which regulate apoptosis. BCL-2 are regarded as protooncogens, promote antiapoptotic processes, and hinder clearance of senescent cells. In vitro and in vivo, navitoclax has shown to inhibit their antiapoptotic activity and promote clearance of senolytic cells. Bussian et al. [30] showed in 2018 that administration of navitoclax in mice that overexpressed p16-positive cells resulted in similar effects as knocking out the corresponding gene. Administration of the substance prevented the upregulation of senescence-associated genes and attenuated tau phosphorylation, an important hallmark of AD.

Dasatinib and Quercetin: Dasatinib is a small molecule and tyrosine-kinase inhibitor, used in leukemia treatment. Quercetin is a polyphenol compound, more specifically a flavonol, which occurs often in many fruits, vegetables, and plants, and is known for its antioxidant and tumor-suppressive properties. Both substances are FDA-approved and safe for use in humans. The substances were selected by Zhu et al. through a mechanism-based approach, as opposed to the random high-throughput screening usually used for drug discovery, harnessing their properties that were already known. Interestingly, dasatinib and quercitin interact very cell-type-specifically, so that they had to be combined to leverage significant results in different cell types (mouse embryonic fibroblast, human endothelial cells) [27,32,33].

Another very interesting finding in studying dasatinib and quercetin is that they seem to exhibit their senolytic potential also when administered intermittently. Despite their pharmacokinetic elimination half-life of a few hours, a single dose of the substance exhibits senolytic activity for at least seven months. The frequency of administration therefore will potentially depend on the conditions that induce cellular senescence [27,32].

Beside the fact that there may be tremendous clinical potential in senolytic drugs, there are also obstacles that have to be cleared before the full therapeutic potential can be harnessed. As there are practically no direct biomarkers of senescent cells, 42 developments of reliable markers are necessary to further harvest the favorable potential of senolytics.

## 5. Current Therapies and Limitations

Regarding current therapy options, battling symptoms caused by the pathogenetic changes of Aβ-plaques and tau tangles currently is state-of-the art, which limits therapy to relatively late stage of the disease in which patients already suffer from dementia symptoms. Apart from supportive care from family and other caregivers, drug therapy options mostly depend on four substances: cholinesterase inhibitors donepezil, rivastigmine, galantamine, and glutamate antagonist memantine [5]. All of the above mentioned substances treat dementia in terms of memory loss and associated symptoms, but fail in slowing down or stopping the progress of the underlying mechanisms of AD. Therefore, it is in question if Aβ-plaques and neurofibrillary tangles alone are a sufficient targets in treating AD.

**Figure 1:**
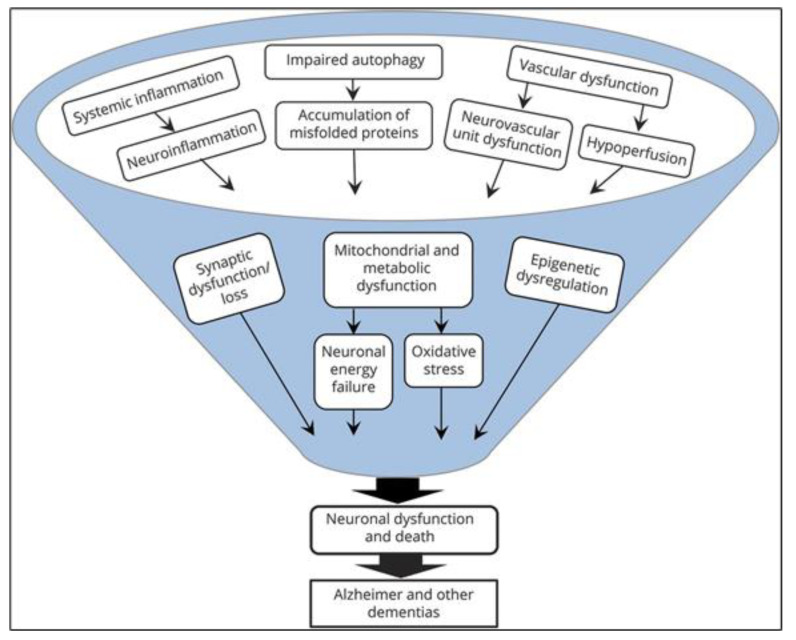
Microglial cells respond differently depending upon type and intensity of activation signals. [17].

Several large phase III trials of anti-amyloid approaches in patients with mild to moderate AD have been published, but the disappointing results contributed to a paradigm shift in the scientific community: Many age-related changes in cellular and other physiological processes are now known that induce or contribute to the formation of Alzheimer’s disease and other neurodegenerative diseases [2,7]. It is now understood that the many different involved mechanisms and pathogenetic hallmarks (Figure 1) demand the investigation of more diverse drug targets and definition of reliable biomarkers, aiming at the long pre-dementia stage of the disease at best. Prolonging the symptom-less phase and preventing the advancement of AD should be the main focus in concepting new therapies.

### Nutraceuticals and AD

The links between diet and human disease are gaining increasing interest among investigators. The term nutraceutical is defined as any food (fruits, vegetables, nuts, tea, and botanical products, among others) or part of a food such as a dietary supplement that produces a medical or health benefit, including the prevention and treatment of a disease. Generally, nutraceutical products are administered in dosages that exceed those that could be obtained from normal foods, considering the absence of very low toxicity to the organism. On the other hand, functional foods are now being examined for their physiological benefits and for their ability to reduce risk factors in chronic diseases. The principal difference between nutraceuticals and functional foods is the customary presentation of the product (nutraceuticals with pills, capsules, beverages, whereas functional foods are those such as whole vegetables, fruit, or natural products) [34].

Epidemiological evidence linking diet, modifiable environmental factors, and the risks of AD is rapidly increasing. A non-balanced diet appears to impact on the risk of AD. There is a belief that the customary diet in Eastern countries has an important contribution to the health of that population as well as for resisting and preventing age-related diseases [35,36].

## 6. EGCG

Epigallocatechin gallate (EGCG), also known as (−)-epigallocatechin-3-*O*-gallate, is a polyphenol compound. Chemically, it consists of the ester of epigallocatechin and gallic acid. As a catechin, it belongs to the group of flavonoids. Regarding neurodegenerative diseases, several in vivo as well as human observational and intervention studies have shown that there is an inverse link between the amount of green tea catechin intake and cognitive impairment, while others did not.

In an AD mouse-model, Rezai-Zadeh et al. showed that high doses of EGCG (administered orally or by i.p. injection) significantly lowered Aβ pathology and plaques and provided a significant cognitive benefit to the affected mice [37]. Scholey et al. [38] conducted a double-blind, placebo-controlled crossover interventional study, which found that 300 mg EGCG administration was associated with a significant increase in α-, β-, and θ-brain wave activities. In a double-blind, randomized controlled study conducted in Japan, participants were given 2 g/day of green tea powder (containing 220 mg of catechins) or for 12 months. However, the results showed that one year of green tea consumption did not significantly affect cognitive function [39].

Additionally, a range of observational studies has been conducted in humans in recent years (see Table 1), with mixed outcomes.

All mentioned studies focused on cognitive functions as a whole and explored whether EGCG/green tea catechins could be a viable substance to combat or prevent a loss in memory function as a standalone therapy.

### 6.1. EGCG and Autophagy

Apart from the antioxidant properties of green tea catechins, their autophagy-inducing effects have been studied in a variety of settings. As mentioned before, the mTOR pathway plays an indispensable role in autophagy activation. Holczer et al. [51] showed in 2018 that ECGC treatment is able to induce mTOR-dependent autophagy in HEK293T (human embryonic kidney) cells. It further weakens the effect of negative regulators of autophagy (such as GADD34) that control apoptosis. Therefore, EGCG was able to extend autophagy, delaying apoptosis mediated cell death and eventually extending cell viability [51,52].

In endotoxin-stimulated macrophages, optimal concentrations of EGCG were able to induce autophagy and anti-inflammatory effects. EGCG achieved this by inhibiting HMGB1 release and stimulating its autophagic degradation [53].

Grube et al. [54] found that ECGC is able to induce autophagous effects on primary human glioblastoma cell cultures. In high but CNS-achievable concentrations (100 µM), the substance (which was applied in pure form as well as a tea extract dietary supplement) activated autophagy in the brain cancer cells and triggered different endogenous repair mechanisms to protect the cells. After treatment with very high levels of EGCG (500 µM), strong induction of autophagy and apoptosis was observed. This confirms a 2008 study by Hashimoto et al. [55], which suggests that higher concentrations of EGCG do not promote but inhibit autophagy and lead to apoptosis. The macrophage cell lines used in this study were treated with 100 µM and above.

Overall, autophagy-inducing properties of EGCG depend upon the dosage used, level of cellular stress, and the cells lines used. When considering available evidence, the correlation between EGCG and autophagy are not yet fully understood. Nevertheless, conducted studies have shown that EGCG possesses autophagous-inducing properties in vitro, and that there is some potential to exert these properties also in vivo as well. To shed light on this, more thorough and especially clinical research will be necessary.

### 6.2. EGCG and Neuroinflammation

**Figure 2:**
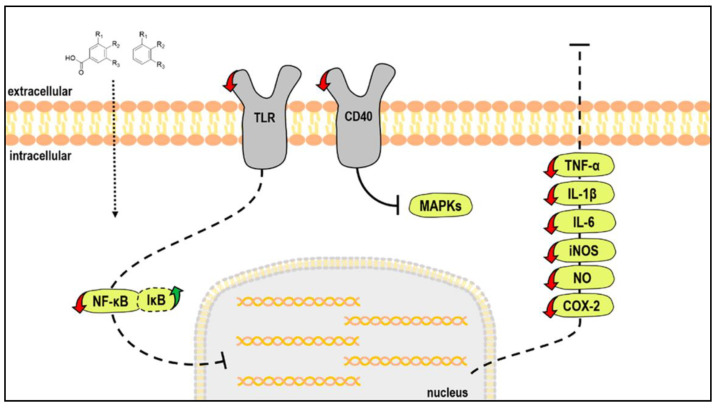
Main molecular targets of low-molecular weight polyphenols metabolites in stimulated microglia cells. [17].

EGCG is a polyphenol and flavonoid compound, both groups of chemicals that have been found to interfere with cascades that promote neuronal inflammation. Studies have shown that there is a link between flavonoid-rich diets and lower levels of inflammatory biomarkers [56]. Poulose et al. proved further that Flavonoids inhibit the production of pro-inflammatory cytokines such as TNF-α, IL-6, and IL-1 in microglial cells, suggesting close involvement in pathways such as NF-κB or MAPK (see Figure 2) [17,57].

EGCG in general inhibits secretion of TNF-α, IL-6, and IL-8 through the attenuation of ERK and NF-κB in HMC cells, which could be beneficial in immune suppression treatment and targeting neuroinflammation [58].

Apart from this, EGCG seems to be interacting with TLRs, especially with TLR4. Byun et al. [59] showed that EGCG downregulates TLR4 signal transduction (at a low concentration of 1 µM) in macrophages, hindering TLR4 expression through 67LR. This way, it inhibits activation of downstream signaling and consequent inflammatory responses (MAPK and NF-κB activation) [18,59]. These findings were recently fortified in vivo, as Seong et al. [60] showed that EGCG suppresses the TLR4-NF-κB signaling pathway in mice and therefore exhibited neuroprotective effects.

Several other in vivo studies have come to similar results. Lee et al. [61] tested the effects of EGCG on neuroinflammation and amyloidogenesis in mice with systemic inflammation. The authors demonstrated that EGCG was able to fight CI induced by lipopolysaccharide (LPS) and neuronal cell death. Moreover, EGCG prevented LPS-induced astrocyte-activation and cytokine expression (TNF-α,IL-1β, IL-6) [62].

Overall, EGCG shows great potential in fighting neuroinflammation by interfering with its initiating cascades, suggesting that EGCG could be considered a therapeutic agent for neuroinflammation-associated AD. Since polyphenols in general, and EGCG in particular, interact with more than one inflammation-triggering compound, there is a great need for controlled trials in humans to explore whether the positive effects shown in vitro and animal studies also manifest in humans.

### 6.3. EGCG and Senescence

In protecting the organism from the negative impact of senescence, two properties play an important role: the ability to suppress premature senescence in cells—e.g., through antioxidant or other DNA-protective measures—and the capability of inducing clearance of already senescent cells.

EGCG is widely recognized to possess antioxidant abilities, based upon its chemical structure (i.e., the presence of phenolic groups). Antioxidants are compounds that protect cells against the damaging effects of reactive oxygen species (ROS). When ROS are generated extensively, the imbalance between antioxidant and oxidant substances can damage DNA and induce senescence or cancer [24,63,64].

Numerous studies have shown that EGCG acts as an antioxidant regarding neurodegenerative diseases, and that EGCG is able to scavenge free radical ions and increase the activity of antioxidant enzymes [64,65]. In aging rats, it increases the activity and level of antioxidant enzymes like superoxide dismutase and catalase and non-enzymic antioxidants like tocopherol, ascorbic acid, and glutathione. Further, it decreased the levels of protein carbonyl, all of which can prevent age-associated oxidative DNA damage [66].

As previous work has mostly focused on the antioxidant abilities, few researchers have addressed the question of EGCGs direct senolytic ability—one notable exception is a study conducted on H2O2-treated senescent preadipocytes. Kumar et al. [67] found evidence of ECGCs senolytic ability in concentrations of 50 and 100 µM, in which it downregulated PI3K/Akt/mTOR and AMPK signaling in the cells and also suppressed ROS, iNOS, Cox-2, NF-κB, SASP, and p53 mediated cell cycle inhibition. It further promoted apoptosis of senescent cells by suppressing the accumulation of anti-apoptotic protein Bcl-2.

Lilja, Haslberger et al. [68] recently found a senolytic activity of EGCG in 3T3 pre adipocytes, when targeting SIRT3, an important senolytic factor that can regulate ROS-activity via different pathways.

### 6.4. EGCG and AD

Conclusively, even if recent discoveries are rather promising, the senolytic abilities of EGCG remain mostly unclear, especially in regard to neurodegenerative diseases and AD in particular. EGCG alone may not be a viable option to treat AD as a standalone therapy. The evidence does not support EGCG as a monotherapy in AD or memory loss and the full picture remains unclear, since there is a lack of suitable and reliable RCTs in humans. Another challenge in using EGCG is the question of bioavailability of the compound in the CNS). Nonetheless, promising studies have been conducted in this regard (such as applying EGCG as a pro-drug), which give a positive outlook on this issue.

Nevertheless, the existing results certainly warrant the further exploration of using EGCG in a therapy strategy involving multiple compounds and targeting more than one hallmark of the disease. Especially recent findings regarding its capability to inhibit neuroinflammation are promising and are solid enough to warrant further application of EGCG against AD and neurodegenerative diseases.

## 7. Fisetin

Fisetin (chemically: 7,3′,4′-flavon-3-ol) is a flavonol compound and a member of the flavonoid group of polyphenols (as EGCG). In 2001, Ishige et al. [69] identified fisetin in a screen for flavonoids that are able to prevent oxidative stress-induced nerve cell death, and subsequently its antioxidant capabilities came into focus. Now, it is available as a dietary supplement in pure form, in doses up to 500 mg, marketed to enhance brain health. In recent years, it has achieved the image of a functional food and is promoted to prolong lifespan and counter various effects of aging. Apart from its antioxidant abilities, fisetin has been studied for its wide ranging effects on a number of key pathways involved in cell cycle regulation, apoptosis, the suppression of inflammation, angiogenesis, and metastasis [25]. In vivo, fisetin has been found to enhance long-term memory in non-AD mice (orally administered, 10–25 mg/kg) [70]. In an Aβ1–42 mouse-model of AD, Ahmad et al. were able to show that fisetin significantly decreased the Aβ1–42-induced accumulation of Aβ, BACE-1 expression, and hyperphosphorylation of tau protein. It reversed synaptic dysfunction and had a favorable effect on different proteins involved in AD pathology. Further, it also suppressed various neuroinflammatory mediators. Ultimately, it improved mouse memory when administered intraperitoneally (20 mg/kg/day for two weeks) [71].

### 7.1. Fisetin and Autophagy

Suh et al. [72] tested the effect of fisetin on different types of human prostate carcinoma cell lines. Treatment of cells with fisetin inhibited mTOR activity and downregulated several carcinogenesis-involved proteins that resulted in loss of mTOR complex-formation. Fisetin also activated the mTOR repressor TSC2 through inhibition of Akt and activation of AMPK. However, they showed that fisetin treatment leads to induction of autophagic-programmed cell death rather than cytoprotective autophagy, as shown by the small interfering RNA Beclin1-knockdown and autophagy inhibitor. As the study author noted, this could be worthy in carcinoma treatment, but may be detrimental in treating neurogenerative diseases like AD.

Another recent in vitro experiment, conducted by Park et al. [73], tested whether fisetin can induce autophagy in oral squamous cell carcinoma. Various concentrations (10–300 µM) of fisetin were applied to the cells, which then induced autophagous processes significantly, but simultaneously increased apoptotic cell processes. When applying fisetin in combination with an autophagy inhibitor (in this case, the substance SP600125), apoptosis was elevated further. In case of oral squamous cell carcinoma, this combination seems very promising—in case of neurodegenerative diseases, however, it is not reasonable to raise apoptosis in this manner.

Kim et al. [74] showed in 2016 that fisetin stimulates autophagic degradation of phosphorylated tau via the activation of transcription factor EB and Nrf2 transcription factors, and that fisetin reduces sarkosyl-insoluble tau levels in mouse cortical cells. Treatment of cortical cells or primary neurons with fisetin resulted in significant decreases in the levels of phosphorylated tau. The researchers found out that the activation of autophagy was carried out using the mTOR pathway. Further, the level of LC3-II (an autophagy marker) and autophagy-related gene (ATG) products in cells treated with 5 µM and 10 µM of fisetin were significantly increased.

In vivo, the effect of fisetin on Pb-induced neurotoxicity in mice was analyzed in a 2019 paper by Yang et al. [75]. Fisetin supplementation increased protein expressions and promoted the Pb-induced autophagy in brains of mice (*p* < 0.05). However, no significant difference in these autophagy-related proteins was found between the control group and the group of 50 mg/kg fisetin treatment.

### 7.2. Fisetin and Neuroinflammation

Regarding the ability of fisetin to inhibit neuroinflammation, numerous in vitro and in vivo studies have been published (Table 2).

In vitro, fisetin exhibits significant effects on multiple inflammatory markers. Its ability to interfere with the release of cytokines and other pro-inflammatory substances has been tested in various cell culture models. Notable examples include HMC-1 mast cells, which were stimulated by activated T-cell membranes, where fisetin suppressed cell spreading and gene expression. The stimulation also induced activation of NF-κB and MAPKs. These activations were suppressed by fisetin [76]. As shown by Zheng et al. [79], it reduced microglial activation and inhibited gene expression of TNF-α, IL-1β, COX-2, and inducible iNOS at both mRNA and protein levels.

Subsequently, researchers were able to confirm the findings in animal models. In an AD-mouse model, intraperitoneal injections of fisetin at a dose of 20 mg/kg/day for two weeks not only showed a favorable effect on Aβ1–42 induced memory deficits, but also suppressed various activated NI markers (p-IKKβ, NF-κB, TNFα,and IL-1β), which suggests a clear neuroprotective effect [71]. In another study, Currais et al. [80] showed that oral administration of fisetin in APPswe/PS1dE9 double transgenic AD mice from three to 12 months of age not only prevented memory deficits, but also increased ERK phosphorylation and reduced p25. Elevated levels of p25 cause disregulation of cyclin-dependent kinase 5 (Cdk5) activity that leads to neuroinflammation and neurodegeneration.

In humans, the efficacy of fisetin supplementation vs. placebo on the inflammatory status in patients with colorectal cancer has been assessed. 18 patients received 100 mg fisetin for seven consecutive weeks. Significant changes were observed in IL-8 concentrations in the fisetin group when compared with the placebo group (*p* < 0.03), which suggests that fisetin could improve the inflammatory status in humans [81].

In a very recent paper by Ates et al. [82], promising results were obtained using a fisetin derivative. The substance, CMS121, is a small molecule derived from fisetin, which is developed by a drug discovery paradigm based on phenotypic screening assays. In a transgenic mice model that mimics neurodegenerative diseases, CMS121 alleviates cognitive loss, modulates lipid metabolism favorably, and reduces inflammation and lipid peroxidation in the brains of transgenic AD mice.

Conclusively, study results regarding the anti-inflammatory properties of fisetin look very promising and warrant further studies. Not many researchers have addressed the question if fisetin also exerts protective qualities on the CNS itself, but in vivo studies show a clear correlation and positive effects on long-term memory. Additionally, in the few human trials that have been conducted, fisetin delivers promising results in regard to neuroinflammation by suppressing inflammatory markers.

### 7.3. Fisetin and Senescence

Fisetin as a plant-derived flavonoid is considered a natural occurring senolytic and has shown its ability to disrupt senescence in multiple studies and experiments. This is especially interesting, as in-depth research of the topic has only gained importance in recent years. Senolytic flavonoids such as quercitin and fisetin act in part by inhibiting BCL-2 family members such as BCL-xL as well as HIF-1a and other senescent cell anti- apoptotic pathway (SCAP) network components. In comparison to other flavonoids, fisetin is twice as potent as quercetin in its senolytic properties [25,28].

In vitro, Zhu et al. [83] showed that fisetin selectively induces apoptosis in senescent (but not proliferating) human umbilical vein endothelial cells (HUVECs). It is not senolytic in senescent IMR90 cells, a human lung fibroblast strain, or primary human preadipocytes, which indicates a strong cell type specificity. Interestingly, the fisetin plasma concentrations achieved in a mouse study (2.7–349.4 µM) were similar or higher than those Zhu et al. found to be senolytic in cultured HUVECs. Thus, fisetin has sufficient bioavailability to reach the site of action in the body [83,84].

Yousefzadeh et al. [85] conducted research on a panel of flavonoid polyphenols, which were screened for senolytic activity in senescent fibroblasts. Fisetin was identified as the most potent, because it reduced senescence in murine and human adipose tissue and demonstrated cell-type specificity. It was consequently tested in aged wild-type mice to determine its effect on senescence markers, age-related histopathology, and other disease markers. When administered late in life, it restored tissue homeostasis, reduced age-related pathology, and extended median and maximum lifespan (fisetin dosage: 100 mg/kg orally).

With recent knowledge, fisetin is increasingly becoming accepted as a potent senolytic agent that exhibits its potential on certain cell types as well as in animals and humans. As many studies have focused on the senolytic abilities of fisetin in general, there are is still a knowledge gap of fisetin in relation to AD and neurodegenerative diseases that will hopefully be closed in the following years.

### 7.4. Fisetin and AD

Recent discoveries show that fisetin has positive effects on a variety of pathways associated with neurodegenerative diseases. While the ultimate effects of fisetin on the CNS in humans are still under investigation, it possesses promising characteristics to become part of a therapy approach involving multiple substances.

In conclusion, recent research shows that fisetin has positive effects on a variety of pathways associated with neurodegenerative diseases. While the ultimate effects of fisetin on the CNS in humans are still under investigation, it possesses promising characteristics to become part of a therapy approach involving multiple substances.

## 8. Spermidine

Spermidine, also called monoamine-propylputrescine, is a biogenic polyamine and intermediate product in the synthesis of spermine (which is also biologically active). It occurs in all living organisms and plays a role in cellular growth. Polyamines are essential for cell growth and tissue regeneration. They bind and stabilize DNA, modulate enzymefunctions, and are required to regulate translation [21]. Because the concentration of spermidine decreases in organisms of age, it has been connected to cellular aging [86].

Spermidine is currently studied as a potential cure for a variety of diseases. As a caloric restriction mimetic, it triggers effects similar to fasting regimens, such as intermittent fasting or caloric restriction, which are well-documented to be beneficial for health and life span extension [21]. Currently, it is available as a food supplement in pure form, where it is advertised as life-prolonging agent. However, there was no (EMEA- or FDA-) approved drug available until now.

The life span extending properties of spermidine have been studied in vivo, where spermidine was able to prolong the lifespan of Drosophila melanogaster fruit flies and that of mice of up to 25%, when administered lifelong [87]. With the Bruneck Study, Kiechl et al. [88] have conducted a prospective population-based study over 20 years (1995–2015) to test the potential association between spermidine content in diet and mortality in humans. The community-based cohort study included 829 participants and was able to show that deaths per 1000 person-years decreased across thirds of increasing spermidine intake from 40.5 to 23.7 and 15.1. The difference in mortality risk between the top and bottom third of spermidine intakes was similar to that associated with a 5.7 years younger age. Further, spermidine is studied for its effects on tumorigenesis, cardiovascular and muscle-related diseases, and metabolic syndromes.

The link between spermidine and AD/cognitive decline has been well established. In numerous animal studies, it has shown that spermidine dose-dependently improves performance in most learning tasks up to a given level, from which on performance declines again [89]. In Drosophila melanogaster fruit flies, spermidine supplementation not only showed significant improvements in short and medium-term memory compared to the control group, but also decreased signs of senile dementia and restored memory performance [86,90].

These findings have subsequently been translated into clinical trials. Pekar et al. [91] were recently able to prove the relation of spermidine to age and memory performance. In Austria, serum spermidine levels were correlated to performance in mental state examinations in 80 elderly adults. In this multicentric placebo-controlled study, the participants received spermidine-enriched food, and results demonstrated a positive correlation between enhanced memory performance and serum spermidine level. The authors therefore suggest the inclusion of spermidine levels as a biomarker in the diagnosis of AD.

The inclusion as a biomarker is further warranted by the findings of Joaquim et al. [92]. The researchers were able to show that in AD patients with mild cognitive impairment, the serum spermidine levels are declined and that dynamic changes in the CNS polyamine levels contribute to the cognitive deficit in AD patients.

Conclusively, there seems to be a link between the formation of neurodegenerative diseases and corresponding spermidine levels in the human body. Whether this relationship is causal or a byproduct of other detrimental processes remains unclear and needs to be studied further.

### 8.1. Spermidine and Autophagy

The capability of spermindine to induce autophagous processes—especially macroautophagy—is suggested from an array of studies conducted in recent years. In 2009, Eisenberg et al. [93] showed in a comprehensive study that spermidine induced autophagy in multiple model organisms, such as yeasts, flies, nematodes, and mammals. The researchers tested if the a priori known life span extending properties were due to the induction of autophagy and confirmed this assumption using genetically modified, autophagy-deficient ∆atg7 fruit flies, in which spermidine exhibited no life-prolonging features (dosage: 0.01–1 mM). Additionally, they concluded that several autophagy-related genes (most significantly ATG7) were upregulated after spermidine treatment, and that spermidine induces the formation of a signaling protein into newly formed autophagosomes. In a follow-up study in mice, Eisenberg et al. [94] displayed that spermidine also extends life-span in mice and additionally exhibits autophagy-dependent cardioprotective features. In humans, the researchers correlated spermidine-rich diets with lower blood pressure, but it is not known whether this correlation is causal or not.

Morselli et al. [95] found that spermidine and resveratrol can stimulate autophagy synergistically, both in cultured human cells and in mice. This was achieved by spermidine without targeting the mTOR-pathway, and thus may have less-harmful side effects than autophagy-activation by EGCG or fisetin. In mice, the effect was measurable using intraperitoneal injected spermidine doses of 50 mg/kg, but there was no pro-autophagic effect in doses of 5 mg/kg.

So far, previous work has been limited to in vitro and in vivo studies. It has not been explored if the beneficial effects found in observational studies and RCTs in humans can be attributed to its pro-autophagic capabilities—which are nevertheless very promising in animals. It is of particular interest to correlate plasma spermidine levels and dietary patterns with the amount of autophagic flux and protein acetylation, which can already be determined. This way, a correlation between spermidine, autophagy, and overall health would be possible.

### 8.2. Spermidine and Neuroinflammation

The research of the anti-inflammatory prospects of spermidine, especially regarding neuroinflammation, have not gained as much attention as its pro-autophagic capabilities. However, in recent years this area has also gained in research interest. There are promising findings available, which show that spermidine (and spermine) suppress the expression of several pro-inflammatory cytokines in various research settings (see Table 3).

Of particular interest are results, that can be connected to low-grade, age-associated inflammatory processes, that are connected to the emergence of neurodegenerative diseases. According to Eisenberg et al. [94] and Zwighaft et al. [100], spermidine and other polyamines play a role in this process as they suppress inflammatory cytokines as TNF-ɑ and others that induce chronic low-grade inflammation. This could be shown when spermidine was orally administered in mice.

In humans, blood spermine levels (the main metabolite of spermidine) inversely correlated with the amount of LFA-1 (a protein that mediates adhesion and migration of leukocytes in immune and inflammatory processes), which further confirms the involvement of polyamines in inflammatory processes [101]. The originating mechanisms could lie in epigenetics, for example age-associated changes in DNA methylation patterns. Another possible explanation could be the findings of Yang et al. that indicate that polyamines possess inhibitory effects on pro-inflammatory transcription patterns, such as NF-κB in macrophages [21,102].

Additionally, spermidine was found to suppress overproduction of ROS and the amount of necrotic cell death, which could be inhibited in yeast and human immune cells by adding exogenous spermidine [93]. When necrotic cell death occurs, intracellular compounds including ROS and cytokines leak out of the cell, resulting in local inflammation.

### 8.3. Spermidine and Senescence

Currently the impact of spermidine on the cellular process of senescence is not well understood. Present research places spermidine, or polyamines as a whole, at the crossroads of autophagy and immune senescence, which contributes to their life-prolonging properties. Despite that spermidine itself does not seem to exhibit senolytic or senostatic capabilities, it seems to have indirect effects on the involved processes: Kibe et al. [103] hypothesized that the previously found enhanced longevity in mice may possibly be traced back to the repression of senescence by increased polyamine concentration in the intestinal tract. Matsumoto et al. [104] also contemplate that spermidine and other polyamines are able to inhibit chronic low-grade-inflammation, and therefore possibly hinder the activation of cellular senescence.

García-Prat et al. [21,105] further explain the indirect senolytic capabilities of spermidine as they describe them as dependent on autophagy in stem cells. Spermidine administration reversed the age-associated decline of autophagy in muscle stem cells (satellite cells) in mice, preventing them from entering a senescent state.

Taking together the available evidence, spermidine does not qualify as a senolytic for itself, partly also because of the lack of reliable biomarkers that could show a connection to the involved processes. Current research only connects it to other cellular processes such as autophagy or the inhibition of inflammation that themselves influence and prevent senescence.

### 8.4. Spermidine and AD

In conclusion and regarding the clinical potential of spermidine, its direct AD-influencing capabilities are very promising, as spermidine supplementation has been proven to be beneficial to counteract memory impairment in humans, even if the relevant pathways are unclear. Spermidine does not qualify as a standalone treatment option for acute AD, as the processes involved seem to be rather slow and low-dose dependent. Nevertheless, in recent years it became more and more apparent that low-dosed, long-term supplementation could be part of a strategy to prevent the formation of AD.

## 9. Discussion and Conclusions

Currently, state-of-the-art therapy options do not prevent the progression and formation of AD. Therefore, the clinical implication is that novel approaches in the prevention of AD and other neurodegenerative diseases are necessary. In this regard, the development and clinical investigation of natural, easily available substances with a low profile of adverse reactions should be of special clinical interest, especially when included in a multi-layered therapy approach to counter AD.

All discussed natural components show interesting activities in clinical relevant aspects of cognitive decline or AD. Whereas findings suggest that none of the substances is a viable option for AD monotherapy, a combination of substances could prove to have beneficial effects. Notably, all discussed compounds also show activities in scientific aspects of longevity, where AD is also a disease closely connected to processes of aging.

## Figures and Tables

**Table 1:** Examples of observational studies on the effect of (green) tea consumption on cognitive impairment. Edited after Pervin et al. [40].

#	Primary Author (Year)	Favorable Effect
1	Kuriyama (2006)	Yes [41]
2	Ng (2008)	Yes [42]
3	Huang (2009)	Yes [43]
4	Feng (2010)	Yes [44]
5	Noguchi-Shinohara (2014)	Yes [45]
6	Mashal (2013)	No [46]
7	Nurk (2009)	Yes [47]
8	Wu (2011)	No [48]
9	Feng (2012)	Yes [49]
10	Wang (2014)	No [50]

**Table 2:** Anti-inflammatory effects of fisetin in various studies.

LoE	Model/Study Population	Mode of Action/Result	Ref.
In vitro	HMC-1 mast cells	Inhibits cell-cell communication, NF-κB and MAPK	[76]
In vitro	Lipopolisaccharide-stimulated mouse macrophages	Suppressed activation of NF-κB and JNK MAPKs, but not ERK	[77]
In vitro	IH-3T3 and KF8 cells	Enhanced and sustained activation of ERK and JNK but not p38 in response to TNFα	[78]
In vitro	BV-2 microglial cells	Reduction of microglial activation, PGE2 and NOS production; downregulation of genes for COX2 and IL-1β	[79]
In vivo	APPswe/PS1dE9 double transgenic AD mice	Prevented memory deficits, increased ERK phosph., decreased oxidative stress, downregulation of p25	[80]
In vivo	Aβ1–42 AD mouse model	Downregulated expression of inflammatory mediators p-IKKβ, NF-κB, TNFα,and IL-1β; hindered Aβ accumulation & tau hyperphosphorylation	[71]
RCT	colorectal cancer patients	Reduction of IL-8	[81]

**Table 3:** Anti-inflammatory effects of spermidine in various studies.

LoE	Model/Study Population	Mode of Action/Result	Ref.
In vitro	Murine BV2 microglia cell cultures	Production of inflammatory markers and cytokines decreased upon spermidine treatment	[96]
In vitro	Lipopolisaccharide-stimulated mouse macrophages	Reduced level of pro-inflammatory mediators and cytokines	[97]
In vitro	THP-1 Monocytes	Increased expression and activation of PTPN2, which suppresses IFN-γ activated inflammatory response	[98]
In vitro	Human THP-1 monocytes	Anti-inflammatory effects and increased expression and activity of PTPN2	[98]
In vivo	Mice with ear edema	Decreased ear thickness, mediators of inflammation markers, and neutrophil infiltrations	[97]
In vivo	Zebrafish *(Danio rerio)*	Prevention of LPS-induced NO production, decreased ROS accumulation, and reduced inflammatory cells recruitment	[99]
